# Overdue to understand anticoagulation in pulmonary arterial hypertension

**DOI:** 10.1177/2045893217743184

**Published:** 2018-03-14

**Authors:** Kishan S. Parikh, Michael P. Gray, Lewis J. Rubin, David Badesch, Richard A. Krasuski

**Affiliations:** 1 3065 Duke Clinical Research Institute, Durham, NC, USA; 2 456984 Pulmonary Hypertension Association, Silver Spring, MD, USA; 3 12220 Division of Pulmonary Sciences and Critical Care Medicine, University of Colorado Anschutz Medical Campus, Aurora, CO, USA; 4 1878 Department of Medicine, University of California, San Diego, La Jolla, CA, USA

Dear Editor:

We read with interest the retrospective study by Roldan et al.^1^ assessing the safety of oral anticoagulation in 201 patients with pulmonary arterial hypertension (PAH). It is remarkable that while approximately 60% of patients with PAH are anticoagulated, consistent with prior data, half of them have a sole indication of PAH and possibly a higher incidence of major bleeding compared to other populations that require anticoagulation. The authors then note in the discussion that “a randomized clinical trial is unlikely to be viable;” we believe that while this statement may be true based on prior efforts, it is unacceptable. A randomized trial of anticoagulation strategies in PAH is urgently needed as an aging PAH population on more medications will continue to accumulate bleeding risk. A reminder to its importance is consideration of patients’ perspectives in this decision. We (KSP, RAK) interviewed 43 patients with PAH at the Research Room at the Pulmonary Hypertension Association’s 2016 International PH Conference and Scientific Sessions (Dallas, TX, USA; June 2016) about their experiences with anticoagulation and found that patients overwhelmingly desired to participate in a clinical study that would answer this question. Of note, several patients were taking direct oral anticoagulants to treat their PAH, for which there are no data to support.

We believe that a PAH population, already taking a median of ten medications and dealing with high morbidity as noted by Roldan et al., must have the ability to make a more informed decision on anticoagulation in conjunction with their providers. While a randomized clinical trial of anticoagulation in PAH has not yet happened, we believe findings such as these, and the expanding armamentarium now including direct oral anticoagulants, should remind us that the need and urgency are seemingly only growing.
